# Status and development for detection and control of ammonium bisulfate as a by-product of SCR denitrification

**DOI:** 10.1038/s41598-021-90040-w

**Published:** 2021-05-17

**Authors:** Kunling Jiao, Xiangyang Chen, Xuan Bie, Daokuan Liu, Mingjie Qiu, Shuangchen Ma

**Affiliations:** 1 Department of Environmental Science and Engineering, North China Electric Power University, Baoding, 071003 Hebei China; 2 School of Energy and Environment, Inner Mongolia University of Science and Technology, Baotou, 014010 Inner Mongolia China

**Keywords:** Environmental sciences, Energy science and technology, Engineering

## Abstract

When denitrification technology using NH_3_ or urea as the reducing agent is applied to remove NOx from the flue gas, ammonium bisulfate (ABS) by-product will also be generated in the flue gas. ABS has an impact on catalyst life span, denitrification efficiency etc., air preheater and its downstream thermal equipment also have a significant negative impact due to its plugging and corrosion. The requirement for NOx removal efficiency is improved by ultra-low emissions in China. However, wide-load denitrification makes the flue gas composition and temperature changing more complicated. Increasing ammonia injection can improve the NOx removal effect, but too much ammonia injection will lead to the formation of ABS and the increase of deposition risk, the contradiction between these two aspects is amplified by ultra-low emissions and wide-load denitrification in many plants. Coordinating NOx control and reducing the impact of ABS on equipment are issues that the industry needs to solve urgently. In recent years, extensive research on ABS had been carried out deeply, consequently, there has been a relatively in-deepth knowledge foundation for ABS formation, formation temperature, deposition temperature, dew point temperature, decomposition behavior, etc., but the existing researches are insufficient to support the problem of ABS under full load denitrification completely resolved. Therefore, some analysis and detection methods related to ABS are reviewed in this paper, and the impact of ABS on SCR, air preheater and other equipment and the existing research results on reducing the impact of ABS are summarized also. It is hoped that this review will provide a reference for the industry to solve the problems of ABS that hinder wide-load denitrification and affect ultra-low emissions.

## Introduction

Coal-fired flue gas contains sulfur oxides and nitrogen oxides, which not only cause a direct negative impact on the environment, but also cause serious environmental problems, such as acid precipitation, photochemical smog and haze, etc.^1^. The selective catalytic reduction (SCR) technology is mature and has high NOx removal efficiency. It is now the mainstream technology for NOx control in the world. This method uses NH_3_ or urea as a reducing agent to reduce NOx in the flue gas into N_2_, but the effects of sulfur oxides and NH_3_ in the flue gas and water vapor in the flue gas will generate ammonium bisulfate^2^ (simply called, ABS).

As the flue gas temperature decreases along the flue gas flow, ABS is initially generated in the temperature range of the SCR. It is an acid salt that can occur a chemical reaction with the metal oxide components in the SCR catalyst, and can also cover the active sites of the catalyst by covering or clogging the micropores of catalyst, resulting in reduced denitrification activity^3–8^. The ABS migrated from SCR or generated in the air preheater adheres to fly ash and deposits on the air preheater, causing the air preheater plugging^9,10^. Part of the ABS reacts with fly ash, which changes the properties of fly ash and affects the efficiency of the dust collector; the generation of ABS will also increase the emission of fine particles from coal-fired power plants^11–14^. So it can be found that ABS has many influences on SCR and downstream equipment.

In recent years, full-load denitrification has been proposed following the ultra-low emission transformation. Ultra-low emission requires high efficiency in controlling NOx removal, and wide-load denitrification requires the denitrification device to operate under the complex conditions. When the flue gas temperature is low, the denitrification efficiency is low, which leads to an increase in ammonia escape. At the same time, to ensure a higher denitrification efficiency, the amount of ammonia injection is often increased, or the ammonia injection is not adjusted in time when the load fluctuates, which may easily cause excessive ammonia injection^15^. More, these factors can all lead to an increase in ammonia escape. The escaped ammonia will promote the production of more ABS, and ABS is an important factor in catalyst blockage and decreased activity. Therefore, in the context of ultra-low emissions and wide-load denitrification, the generation of ABS is more serious, and the increase in ABS generation is bound to aggravate its negative effects. Therefore, it is urgent to carry out in-depth research on the related properties of ABS and adopt appropriate methods to control its impact.

The research team has reviewed the research on the formation, decomposition, migration, and deposition characteristics of ABS^16^. This article will review the collection and detection methods of ABS-related substances and the impact of ABS (including the effects of SCR denitrification, catalyst, air preheater, and fly ash properties etc.). To help relevant personnel in the industry improve their understanding of ABS, advance ABS research and promote the resolution of ABS problems.

## ABS collection and detection methods

Choosing an appropriate method of ABS collection and analysis is a necessary basis for in-depth research on ABS characteristics. Due to the different focus of research on ABS by related scholars, their collection, analysis and detection methods are also different. For example, research on ABS generation requires collection or detection of ABS, research on ABS decomposition requires collection and detection of its decomposition products. The same goes for studying volatilization and decomposition. When collecting ABS, the quartz tube can be used as the deposition medium, and then the quartz tube can be filled with quartz wool, or glass beads can be used as an auxiliary to improve the collection effect. Research by Xi’an Thermal Power Research Institute in China found that the spiral quartz tube can achieve a good ABS condensation collection effect by improving the temperature field and flow field^17^.

When studying ABS-related issues in SCR, the most common detection method is FT-IR(Fourier transform infrared spectroscopy) to observe the characteristic absorption peak of ABS. This method can only be qualitative but not quantitative. In order to quantitatively analyze the ABS deposited on the catalyst surface, Yang crushed the catalyst, weighed a certain mass, added sodium hydroxide, heated to evaporate and then absorbed ammonia with a dilute sulfuric acid solution, and finally measured the ammonium concentration by spectrophotometry^18^. However, due to the complexity of the composition of the ammonium sulfate salt, the results obtained by only measuring the ammonia content cannot accurately represent the ABS content on the catalyst surface.

Shi analyzed the Raman spectra of ABS, (NH_4_)_2_SO_4_(AS), (NH_4_)_3_H(SO_4_)_2_, and found that the characteristic peaks of the three overlapped more and it was difficult to distinguish by optical method^17^. XRD(X-ray diffraction) is often used for the qualitative analysis of the composition of sedimentary products. XRF( X-ray fluorescence spectrometer) and EDX(Energy dispersive X-ray spectroscopy) elemental analysis can quantify the N and S elements, but the composition of the sediments is complex and the species composition and content cannot be determined only by elemental analysis. Therefore, it is a more accurate method to measure NH_4_^+^ and SO_4_^2−^ separately by chemical methods and distinguish the types of ammonium sulfate based on the molar ratio.

Zhi used TDLAS(Tunable diode laser absorption spectroscopy) to detect NH_3_ produced during the decomposition of ABS, but the absorption intensity of characteristic frequency light by ammonia was greatly affected by temperature changes^19^. Yang et al. used a bubble absorption bottle containing dilute sulfuric acid and a controlled condensation method to collect NH_3_ and SO_3_ in the actual flue gas, and analyzed the concentration of SO_4_^2−^ and NH_4_^+^ ions by thorium reagent spectrophotometry and indophenol blue spectrophotometry^18^. Zheng achieved a good collection effect by filling the collection device with glass beads, and established an evaluation reaction product composition system with NH_4_^+^ and SO_4_^2−^ (denoted as N, M, respectively) as analytical indicators in the NH_3_ and SO_3_ reaction experiments^20^. As shown in Table 1.

**Table1:** Composition analysis method of ammonium sulfate and ammonium bisulfate.

The relationship between M and N	Product composition
M ≥ N	The product is only ammonium bisulfate, the total amount is N
M ≤ 0.5 N	The product is only ammonium sulfate, the total amount is 0.5 N
0.5 N < M < N	Products are N–M ammonium sulfate, 2 M-N ammonium hydrogen sulfate

Liu et al. summarized the collection and detection methods of SO_3_^21^. Zhang Yu introduced the detection methods of NH_3_ in fly ash and proposed optimization suggestions^13^. Zhou gave the titration method for detection of SO_3_ and SO_2_^22^.

Qu reviewed the collection and detection methods of SO_3_ after SCR^23^. Shi classified ABS-related detection technologies into three categories: gas phase ABS, ABS in ash, and ABS reactant detection, and gave a detailed description and summary^17^.

There is still room for improvement in the collection and detection and analysis of substances in the study of ABS characteristics. This article summarizes some of the collection and analysis methods of ABS-related species such as ABS, SO_2_, SO_3_, and NH_3_.Tables 2 and 3 can provide references for subsequent researchers' experimental system design.

**Table 2:** Collection methods.

Detection object	Sampling method	Detection Indicator
SO_3_(g)	Controlled condensation^24^	SO_4_^2^^−^
	Spiral tube method	
	80% isopropanol absorption method	
	NaOH absorption method	
	Direct spectroscopy	SO_3_
NH_3_(g)	Power Plant CEMS(Continuous emission monitoring system)	NH_3_
	Ammonia infrared analyzer	
	Lye absorption	NH_4_^+^
NH_4_HSO_4_	Quartz wool	
	glass bead	
	Electric scale low voltage impactor^25^

**Table 3:** Analysis methods.

Analysis object	Analytical method	
SO_4_^2−^(aq)	Spectrophotometry	
	Titration	
	Ion chromatography	
SO_3_(g)	Infrared spectroscopy	
SO_2_(g)	Flue gas analyzer, SO_2_ analyzer^26^, infrared, MS (Mass spectrometry)	
NH_4_^+^(aq)	Ammonia ion concentration meter^27^	
	Nessler's reagent spectrophotometry	
	Indophenol Blue Spectrophotometry^28^	
HSO_4_^−^(aq)	Same as SO_4_^2−^ after adding acid	
NH_4_HSO_4_	Measure ammonium and sulfate after dissolution	
	Infrared	NH_4_^+^ 1400,1638 cm^−1^ ^29^, 1451^30^
		HSO_4_^−^ 1174 cm^−1^ ^29^

## Analysis of the influence of ABS on thermal equipment

### 1The effect of ABS on denitrification

ABS comes from the reaction of sulfur oxides and NH_3_ in the flue gas. ABS will affect denitrification through its direct reaction with NO and its interaction with the catalyst. At the macro level, the impact of ABS on denitrification is reflected in the changes in denitrification efficiency.

Yu et al. reviewed the poisoning effects of SO_2_ and H_2_O on commercial vanadium tungsten titanium denitrification catalysts, and believed that ammonium sulfate gradually deposited on the strong and weak adsorption active sites of the catalyst, which would partially deactivate the catalyst; Although a small amount of ammonium sulfate deposition can form new active sites and increase Brønsted acid sites, thereby increasing the denitrification activity, as the amount of deposition increases, the adsorption capacity of newly formed acid sites and active sites decreases, and ammonium sulfate deposits and covering a considerable part of the active sites, resulting in reduced surface active sites on the catalyst and reduced denitrification activity^31^.

**Figure 1:**
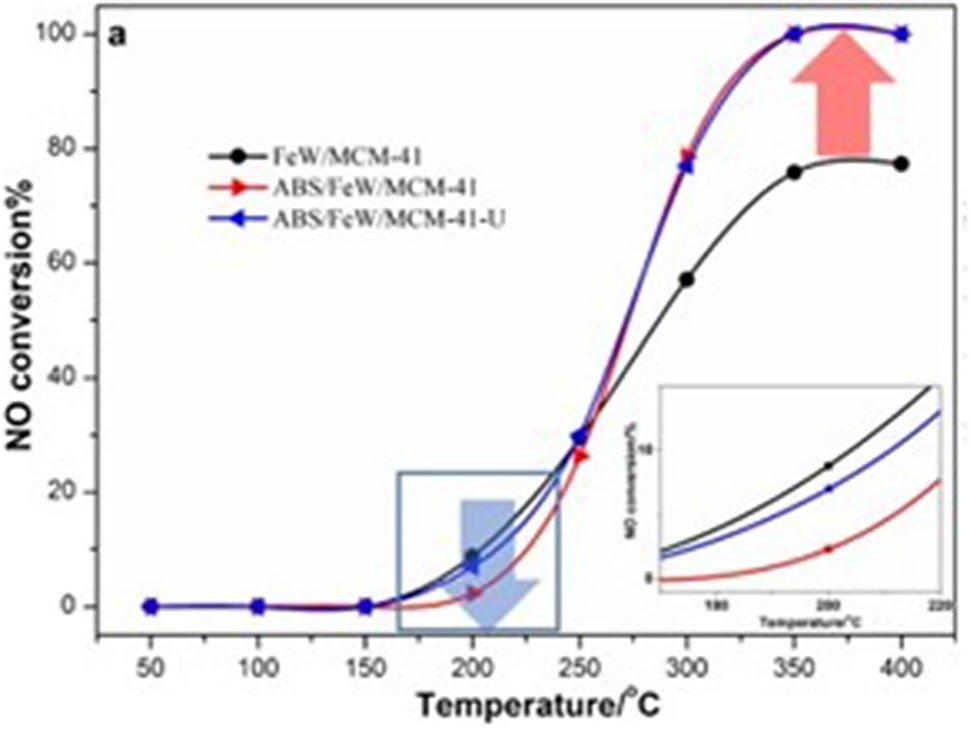
The influence of ABS on FeW/MCM-41denitrification efficiency.

Guo et al. studied the effect of ABS on FeW/MCM-41 catalyst and found that ABS has a dual effect on the denitrification efficiency in different temperature ranges^30^. Above 250℃, it can promote denitrification to a certain extent, as shown in Fig. 1.

**Figure 2:**
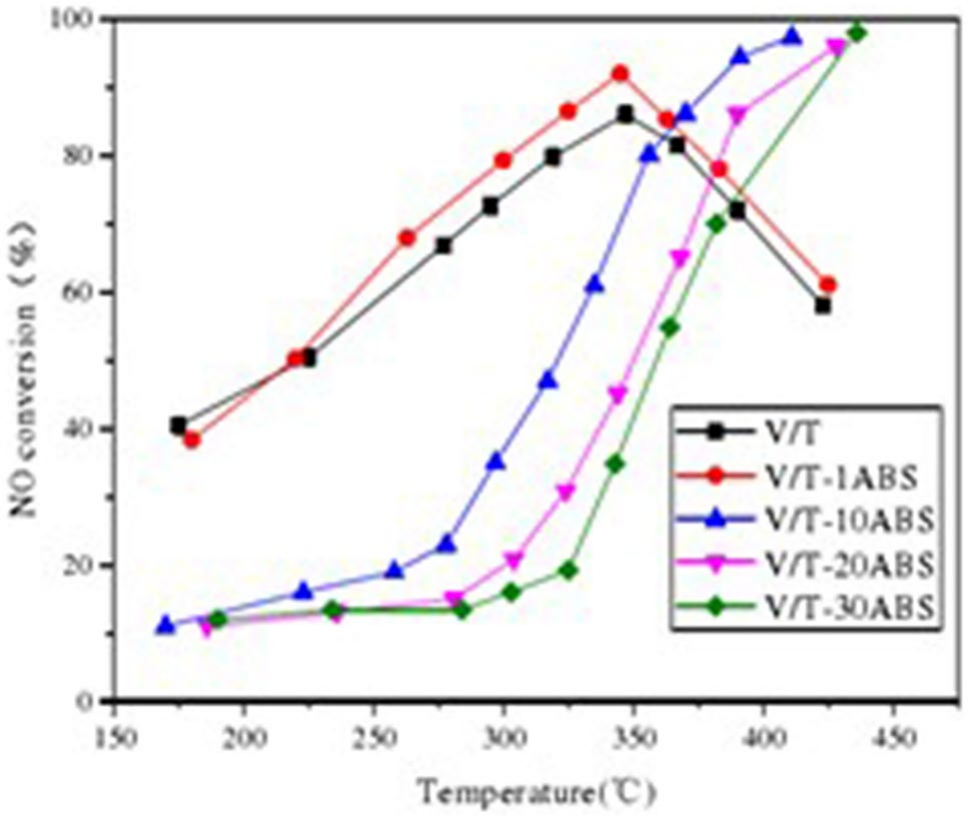
The influence of ABS on V-Ti catalyst denitrification efficiency.

Since the V-Ti catalyst does not have good resistance to ABS poisoning at 350℃, ABS will reduce its denitrification efficiency, as shown in Fig. 2.

It can be seen that ABS has a complex effect that can inhibit denitrification and promote denitrification .The specific performance is: when the flue gas temperature is low, the ABS covering the active sites of the catalyst will cause the denitrification activity to decrease, but when the temperature rises to a certain value, the NH_4_^+^ in ABS can be used as a reducing agent to reduce NOx, and the sulfate ion staying on the catalyst surface as a new B acid acidic site will promote the adsorption and activation of NH_3_^32^.When the amount of ABS deposition is small, it may promote denitrification. When the amount of deposition is large, it will block the micropores and cover the active sites of the catalyst, which will reduce the denitrification activity^5^. In addition, ABS will also be deposited in the ammonia injection control system, which affects the precise control of the amount of ammonia injection.

### The effect of ABS on catalyst

Exploring the reasons for the influence of ABS on denitrification , it can be found that ABS will affect different denitrification catalysts through physical or chemical effects.

The physical and chemical effects of ABS on the catalyst are shown to change the SCR denitrification performance, specific surface area and pore volume, acidity and alkalinity and redox ability of the catalyst. These properties can be characterized by SCR denitrification activity test, BET(Brunner-Emmet-Teller) test, NH_3_-TPD(Temperature programmed desorption), H_2_-TPR(Temperature programmed reduction) or NO + O_2_ DRIFTS(Diffused reflectance infrared fourier transform spectroscopy) and other methods, as shown in Table 4.

**Table 4:** Research methods of ABS influence on catalyst.

Catalyst properties	SCR activity	Specific surface area and pore volume	Acidic	Electron transfer status	Redox
Analytical method	Denitrification activity test	BET characterization	NH_3_-TPD	XPS(X-ray photoelectron spectroscopy)	H_2_-TPD; NH_3_ and NO oxidation; DRIFTS of NO + O_2_^30^

Zhang performed BET characterization of the ABS-loaded SCR catalyst prepared by the dipping method, and found that ABS is more likely to block small pores, and gradually block large pores with increasing loading^5^.

Wang et al. measured the BET and pore structure of the SCR catalyst after SO_2_ and O_2_ sulfidation, and found that the specific surface area and pore volume of the catalyst decreased, but the average pore diameter increased^3^. Combined with the wireless correlation between the activity change and the specific surface area change shown in the catalyst activity test, and the phenomenon that the denitrification activity of the catalyst has increased at higher temperatures. It is speculated that ABS will affect the surface chemical properties of the catalyst, such as the redox ability and surface acidity of the catalyst, thereby changing the denitrification performance of the catalyst. The NH_3_-TPD results also confirmed the effect of sulfurization on the surface acidity of the catalyst.

**Figure 3:**
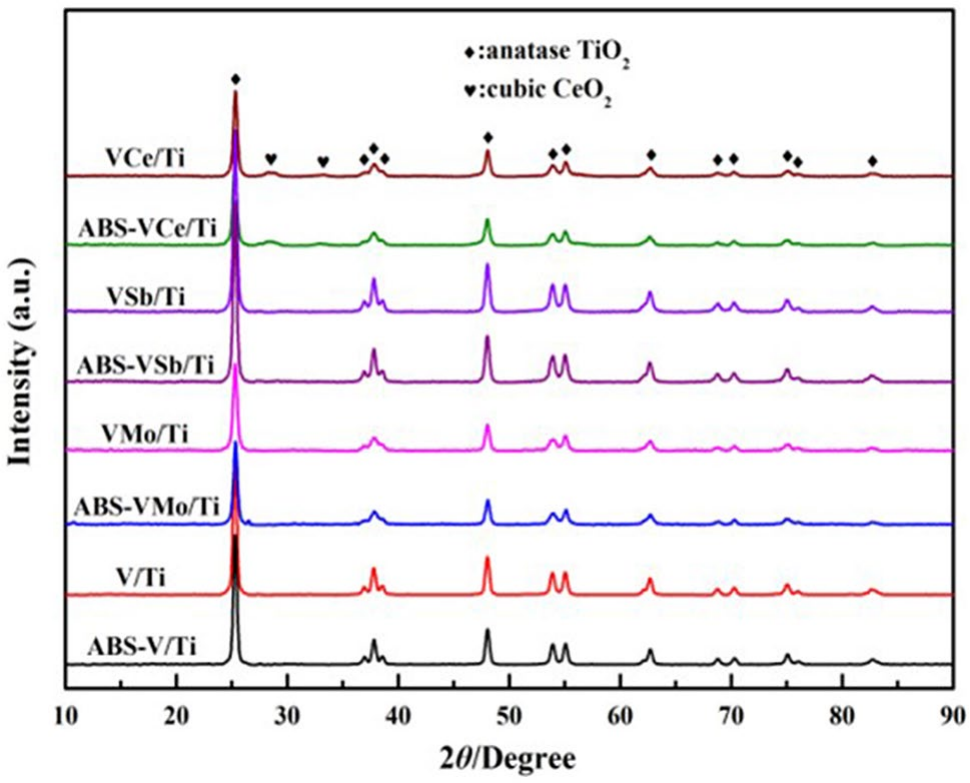
XRD spectra of different catalysts before and after loading ABS^4^.

**Figure 4:**
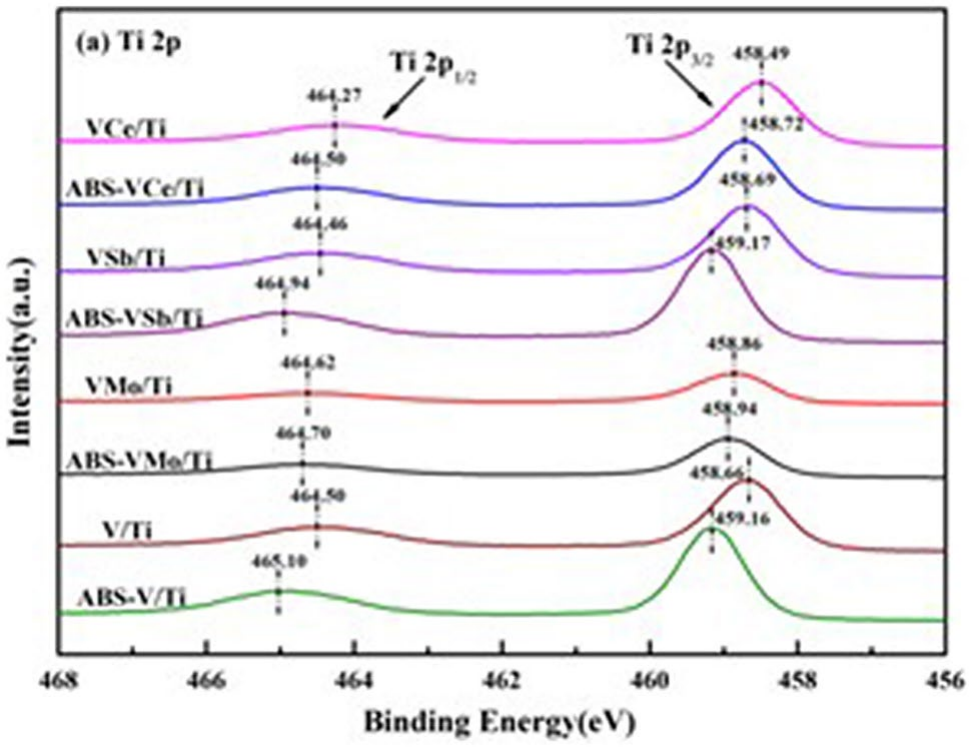
XPS spectra of Ti 2p orbitals of catalyst before and after loading ABS^4^.

Gao compared the XRD spectra of the catalyst loaded with ABS and the fresh catalyst, and concluded that ABS has no effect on the crystal structure of the catalyst^4^, as shown in Fig. 3. But the BET test shows that ABS leads to a decrease in the specific surface area and pore volume of the catalyst, and the XPS results (see Fig. 4) show that loading ABS will reduce the electron cloud density of V, W, Ti, and O elements, and the disappeared electrons are biased toward S of ABS. This indicated that there is a chemical interaction between ABS and catalyst^4^.

The temperature at which the ABS loaded on the catalyst decomposes and releases SO_2_ is around 500℃, and the denitrification temperature window is usually 300 ~ 400℃. In this temperature range, the sulfuric acid produced by the decomposition of ABS can be adsorbed on the surface of the catalyst and caused the catalyst to poison and block^33^. Wang et al. believed that the inhibitory effect of ABS on catalyst activity is reflected in the reaction with Ti to generate TiSO_4_. SCR activity is reduced compared with the TiO_2_ catalyst without ABS deposition^7^.

### The influence of ABS on air preheater

In 1982, J.M.Burke et al. pointed out that it is a solid when only AS is generated in the air pre-heater, and when ABS is generated or AS and ABS are generated at the same time, there will be a two-phase coexistence as solid-liquid^34^.

NH_3_/SO_3_ ratio affects the composition of ammonium sulfate. Under different ratios of NH_3_/SO_3_, low NH_3_/SO_3_ ratio promotes air preheater ash deposition more significantly^11^. Due to the strong viscosity of liquid ABS, it adheres to the heat exchange surface and adsorbs fly ash, which cannot be removed by ordinary soot blowers^35^. The dust accumulation area in the air preheater is usually divided into ABS dust accumulation area and ordinary dust accumulation area. ABS deposition zone temperature is 147 ~ 220℃^36^.

The adhesion of dust includes van der Waals force, capillary force, and Coulomb force^36^. The deposition of ABS in the air preheater is affected by fly ash, temperature field, NH_3_/SO_3_, ash-sulfur ratio, etc., which is more complicated. Luo Min reviewed domestic and foreign research on boiler heating area ash^36^. By constructing the collision and adhesion model between the soot particles and the surface of the heat storage plate, and based on the speed ash accumulation criterion, the ash accumulation model of the air preheater after the SCR denitrification transformation was established. This model can effectively separate the ordinary dust accumulation area and ABS deposition area of the rotary air preheater. Wang Chengyu introduced the fouling model and used Fluent to carry out a software simulation study on fouling characteristics^10^.

Liang believed that mixing ABS in fly ash will increase the thermal conductivity of fly ash, and the reason is that ABS increases the particle size of fly ash^37^. Since the study is the influence of ABS on the properties of fly ash, the increase in fly ash deposition and thickness caused by ABS is not considered. This abnormal result is also explained in the analysis and research results. It can be attributed to the fact that although ABS causes an increase in the thermal conductivity of fly ash, it also makes the fouling situation more serious, so it still causes the heat transfer efficiency of the air preheater to decrease.

### Effect of ABS on the properties of fly ash

**Figure 5:**
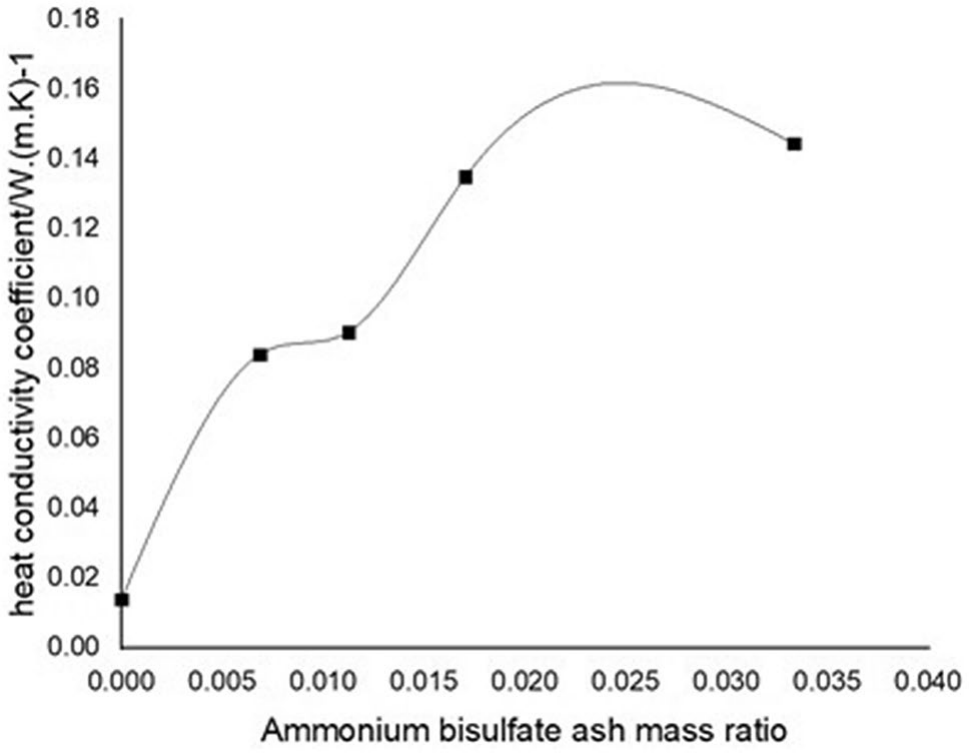
Variation curve of fly ash thermal conductivity under different ammonium bisulfate content^37^.

**Figure 6:**
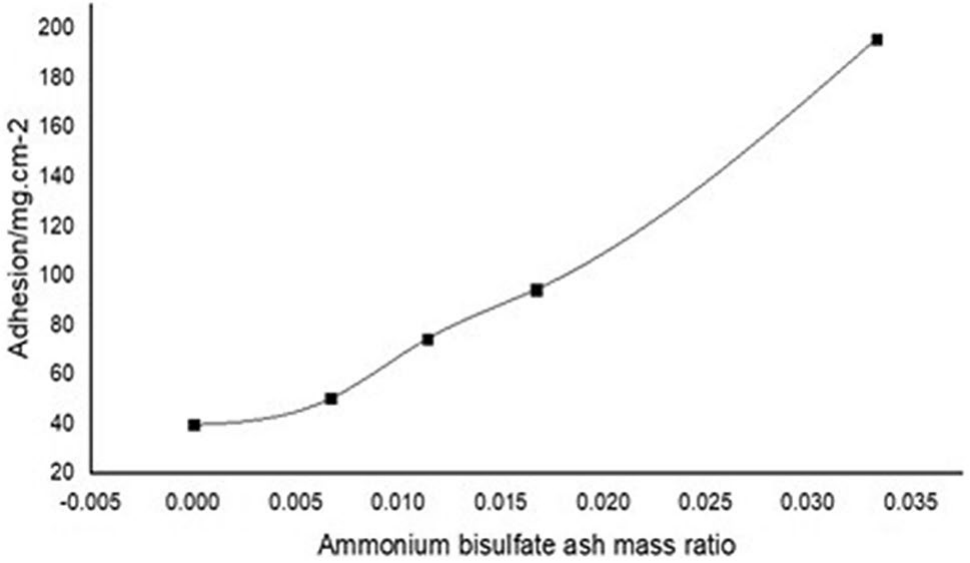
Change curve of fly ash adhesion under different ammonium bisulfate content^37^.

The influence of ABS on the properties of fly ash is mainly reflected in the thermal conductivity and adhesion. Liang Dengke introduced the concept and testing technology of fly ash adhesion and the concept and testing technology of thermal conductivity^37^. By mixing ABS with the fly ash collected by a dust collector without denitrification, and preparing an experimental ash sample by stirring and heating, the study found that ABS can increase the particle size of fly ash (shown in Fig. 5), increase moisture absorption, and adhesion Enhancement (shown in Fig. 6). The more ABS in ash, the more obvious its effect. Due to the increase in particle size caused by doping with ABS, the reduction of fly ash specific resistance is beneficial to dust removal^38^, which is one of the beneficial effects of ABS. However, the increased adhesion makes it difficult to clean the electrode plate, and the increase in moisture absorption means that the deposition of ABS on the air pre-heater will increase the risk of corrosion.

Wang conducted a TG-DSC(Thermogravimetric-Differential scanning calorimeter) test on fly ash added with ABS, and found that ABS had an effect on the metal oxides in the fly ash, so that the fly ash could be partially melted in the air preheater^10^.

Lu et al. indicated that the flue gas temperature in the air preheater is usually 15 ~ 45℃higher than the wall temperature, which means that when the flue gas temperature is higher than the condensation temperature of ABS^39^. The wall temperature may be lower than the condensation temperature to cause ABS deposition, and the presence of fly ash will aggravate the deposition of ABS. The study also found that ABS adheres to small particle size fly ash more obviously.

### The impact of ABS on fine particulate matter emissions

**Figure 7:**
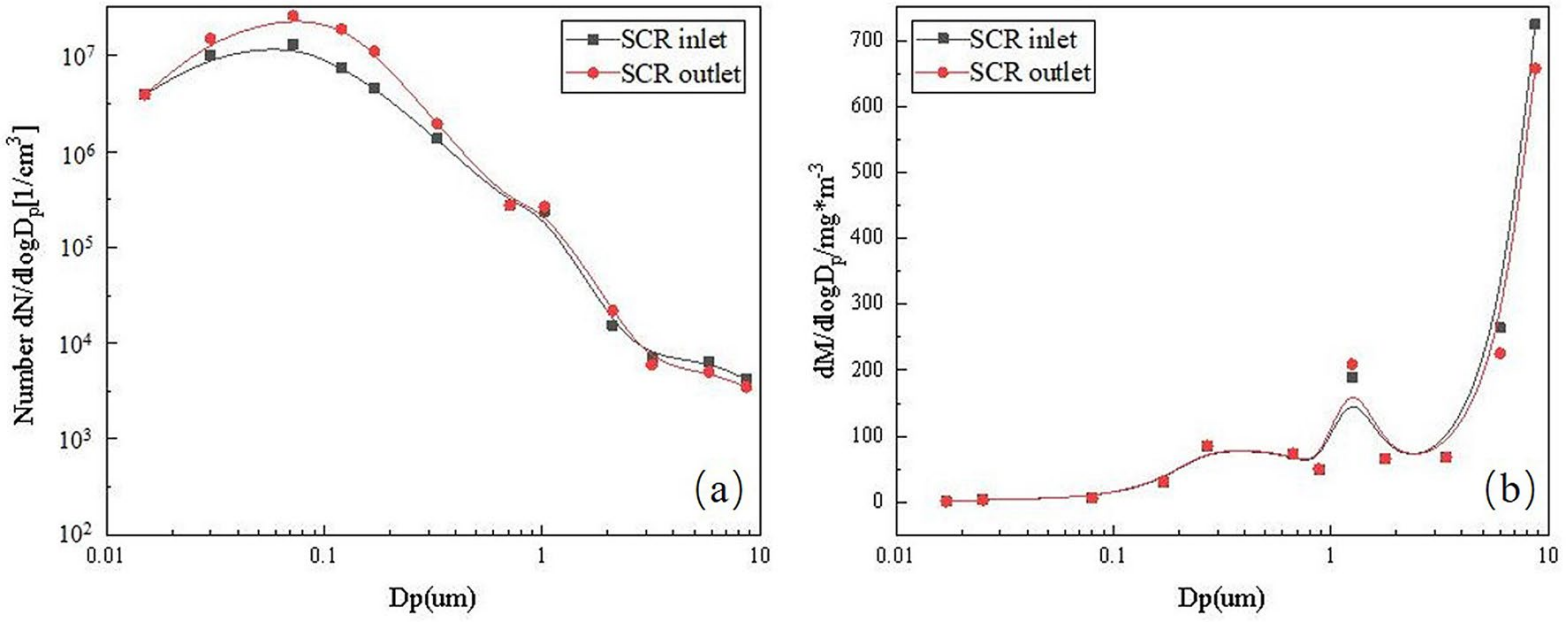
Changing in particulate matter at inlet and outlet of SCR reactor^12^ (**a**: Number concentration, **b**: Mass concentration).

NH_3_, SO_2_, SO_3_, H_2_O, O_2_ in the flue gas will increase the emission of fine particles after entering the SCR denitrification. The fine particles are mainly the Aitken core mode with a particle size of less than 0.08m^40^ and submicron particles with a particle size of 0.02 ~ 1 μm(as shown in the red curves in Fig. 7(a))^12^. These submicron particles are mainly composed of ABS and a small amount of AS^12,25^. From the point of view of the mass concentration of fine particles at the inlet and outlet of the SCR reactor (as shown in Fig. 7(b)), the change trend is not obvious, and the micron-sized particles with larger particle size still account for a higher proportion. Part of the SO_3_ and ammonium sulfate fine particles will be discharged with the flue gas from the SCR denitration system. Although the dust removal efficiency of the existing ESP of coal-fired power stations can be as high as 99%, but the capture rate of PM_2.5_ is low, so it cannot effectively remove the fine particles formed in the SCR denitration process^40^. Even though WESP can remove a part of SO_3_, its ability to remove fine particles is poor. The final ammonium sulfate fine particles discharged from the flue gas treatment system will increase^12,31^.As a result, the installation of SCR reduces NOx emissions and NOx-related secondary pollution while increasing the emissions of ammonium sulfate particles.

### The influence of ABS on the properties of fly ash

Ammonium sulfate will be partially deposited on the surface of the equipment as the flue gas flows. When cleaning by sonic soot blowing, the fly ash on the equipment surface enters the dust removal system together with other fly ash, and eventually it will be unevenly dispersed in the raw fly ash collected by the dust collector. As the ash cleaning system regularly cleans the ash, the final ammonium sulfate will be unevenly dispersed in the fly ash. This unevenness will cause the local ammonium sulfate ratio to be too high. This situation is more serious when ammonium sulfate is formed and more deposited on the surface of the equipment, resulting in increased acidity of fly ash and increased particle agglomeration^13^.

## ABS control method

Different scholars have carried out research on improving the catalyst's anti-ABS clogging ability and low-temperature denitrification activity from the perspectives of catalyst modification, regeneration, SCR and air preheater operation, and ABS generation precursor control. It is a good strategy to control the oxidation of SO_2_ by the SCR catalyst to reduce the generation of SO_3_, thereby achieving the purpose of controlling the generation of ABS^41^. Chen et al. intercalated the NH_4_^+^ of ABS into the interlayers of MoO_3_, leading to a NH_4_^+^-HSO_4_^−^ cation–anion separation by conquering their strong electrostatic interactions. Subsequently the separated NH_4_^+^ was consumed by taking part in low-temperature NH_3_-SCR. Meanwhile, the surface HSO_4_^−^ separated from ABS oxidized the reduced catalyst during the NH_3_-SCR redox cycle, concomitant with release of SO_2_ gas, thereby resulting in decomposition of ABS^42^. Li et al. believed that inhibiting the formation of ammonium sulfate or promoting the decomposition and transformation of ammonium sulfate at low temperature is the core of low temperature denitrification catalysts against SO_2_ poisoning^43^. Zhang believed that the anti-ABS clogging performance of the catalyst mainly depends on the balance of condensation and decomposition of ABS^5^. Vanadium oxide can promote the decomposition of ABS on the catalyst surface, but too high vanadium oxide will increase the SO_2_ oxidation rate. Therefore, to solve the problem of ABS deposition, an auxiliary agent that reduces the decomposition temperature window of ABS and ensures the oxidation rate of SO_2_ is needed. Wang et al. believed that the sulfur resistance of the catalyst can be improved by enhancing the reaction between ABS and NOx^7^. During the full load process, ABS/AS is deposited on the surface of the catalyst in the low temperature section, and when the temperature rises, the deposits on the surface gradually decompose and return to the flue gas^4^. Żyrkowski et al. believed that ABS deposition can be reduced by controlling the SCR operating temperature at 300 ~ 400℃^44^.

### SCR catalyst modification

Researchers have carried out investigations on improving the low-temperature activity of catalysts, resisting ABS poisoning ability, reducing SO_2_ oxidation rate and ABS decomposition temperature, etc. for different catalyst active components, carriers, and active additives.

The V_2_O_5_/AC(Activated carbon) catalyst with activated coke as the carrier has a good low-temperature resistance to SO_2_ poisoning because the carrier AC has a strong low-temperature reduction ability to H_2_SO_4_^43^. Gao Lei found through thermogravimetric experiments that the decomposition temperature of ABS decreased with the addition of WO_3_^4^. XPS results show that the addition of WO_3_ to the catalyst can promote the decomposition of bidentate sulfate on the catalyst surface into SO_2_ because it increases the electron cloud density of S. Li et al. indicated that the addition of WO_3_ in the V/W/Ce/Ti-5% catalyst changed the alkalinity of the catalyst and weakened the chemical reaction between ABS and the catalyst^45^.

MoO_3_, Sb_2_O_3_, CeO_2_ and other active additives can be added to the V_2_O_5_/TiO_2_ catalyst. Among them, MoO_3_ has the best effect on the decomposition and reaction behavior of ABS. The addition of CeO_2_ will result in the formation of Ce_2_(SO_4_)_3_ which is more difficult to decompose during the decomposition of ABS, which is not conducive to the decomposition of ABS^4^.

FT-IR, DFT(Density functional theory) and XPS showed that the active materials and additives of the V-5Mo/T catalyst function as a protective layer to prevent sulfur species from forming strong bonds with Ti, thereby promoting the decomposition of ABS. V, W, and Mo all have the function of protecting ABS from forming a strong bond with Ti, thereby promoting the decomposition of ABS. However, the V-5Mo/T catalyst has a good denitrification activity due to the NH_3_ absorbed by the Lewis acid site is greatly increased, and can promoted the reaction of NO and NH_4_^+^ of ABS and the decomposition of ABS released by SO_2_^5^.

Chao found that doping with rare earth elements can improve the low-temperature reaction activity and SO_2_ resistance of the SCR catalyst V_2_O_5_-MoO_3_/TiO_2_^46^.In addition, by comparing the decomposition characteristics of ABS loaded on V_2_O_5_-MoO_3_/TiO_2_ and V_2_O_5_-TiO_2_, it is found that MoO_3_ will inhibit the release of SO_2_ and is not conducive to the complete decomposition of ABS. And Ce doping can lower the temperature at which ABS completely decomposes and promote the decomposition of ABS.

**Figure 8:**
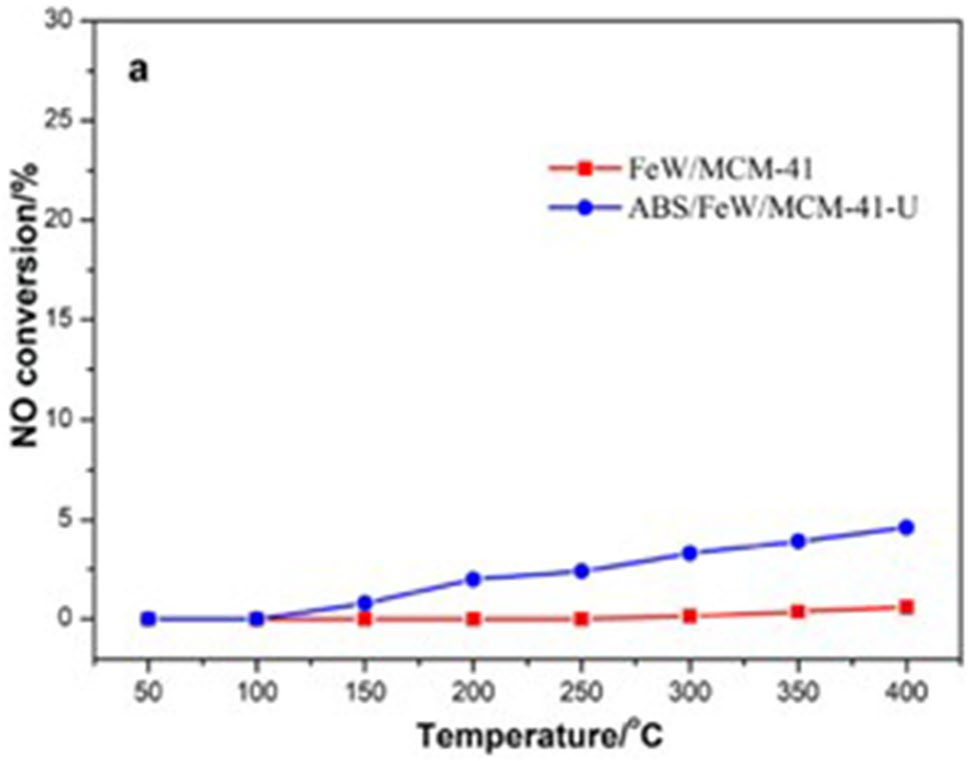
NO oxidation performance test of FeW/MCM-41 catalyst before and after loading ABS^30^.

Guo et al. found that FeW/MCM-41 enhanced the ability to oxidize NO and had better acidity^30^. The improvement of these properties promoted the consumption of NH_4_^+^ in the ABS with a rapid SCR reaction, and promoting the conversion rate of NO, as shown in the blue curves in Fig. 8. In addition, the use of MCM-41 advances the ABS dual-action transition temperature to improve the ABS tolerance of the catalyst.

**Figure 9:**
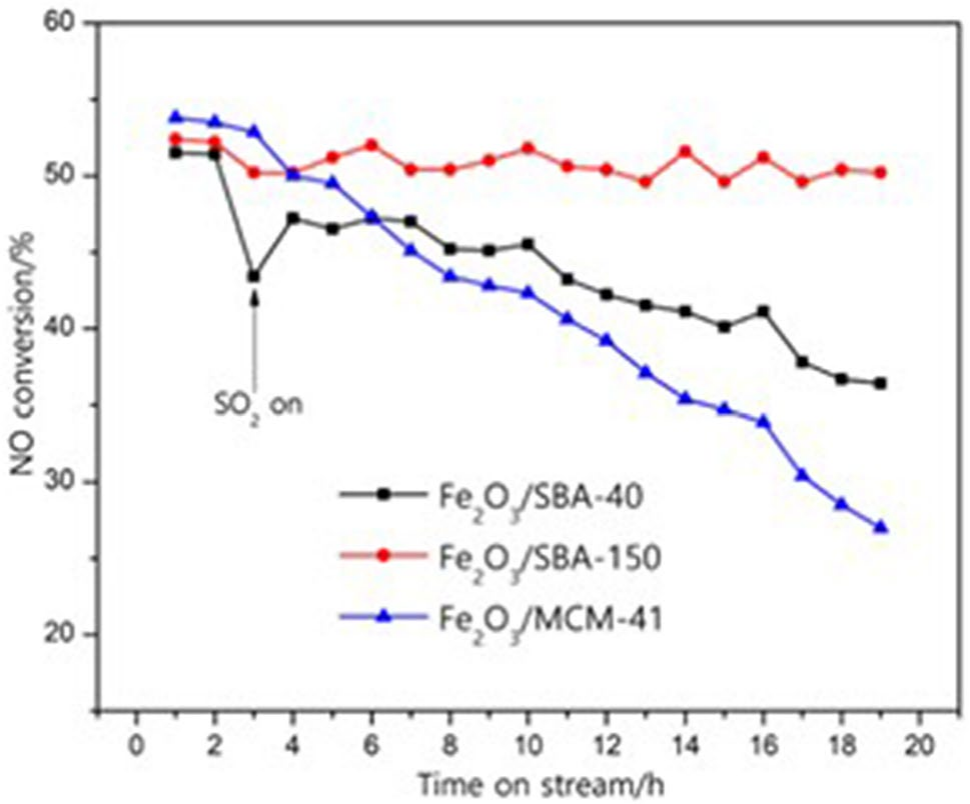
Sulfur resistance tests of catalyst^48^.

Chen et al. found that Fe_2_O_3_-MoO_3_ catalyst has good anti-ABS toxicity because Mo overcomes the electrostatic force of NH_4_^+^ and HSO_4_^−^, has good SCR activity of NH_4_^+^ at low temperature, and HSO_4_^−^ reduces and releases SO_2_ through the study of MAS NMR spectra (nuclear magnetic field) for ^1^H^47^. Guo et al. found that increasing the pore size of Fe_2_O_3_-SBA catalyst can reduce the decomposition temperature of ABS and improve the anti-sulfurization performance of the catalyst^48^, as shown in the red and black curves in Fig. 9, where Fe_2_O_3_/SBA-150 has a larger pore size than that of Fe_2_O_3_/SBA-40.

The addition of Fe_2_O_3_ to the catalyst can inhibit the deposition of ABS on the surface of the catalyst, thereby improving the low-temperature sulfur and water resistance of the catalyst^49^.

Qu researched a catalyst with a wide temperature denitrification window and developed a Ce-Fe-Nb composite oxide catalyst. It has been industrially used in Wenzhou Power Plant, which has certain advantages compared with traditional commercial V-W/Ti catalysts^50^. Li et al. found that the V/W/Ce/Ti-5% catalyst has better low-temperature reaction activity, and promotes the NH_3_-SCR of ABS and NO to reduce ABS production and resistance to ABS poisoning^45^.

Modifying the V-W/Ti catalyst of commercial SCR to improve its sulfur resistance, widening the denitrification temperature window and improving the ability to promote ABS decomposition are powerful measures to achieve wide-load denitrification. Studies have shown that the active component of the catalyst is changed to iron-based copper-based, the active auxiliary is changed to Mo, and the carrier is AC. The use of Nb, Ce doping or MCM-41 molecular sieve catalyst has a good effect on improving the ABS resistance of the catalyst.

### Catalyst regeneration

Catalyst regeneration methods include water washing regeneration, thermal regeneration, thermal reduction regeneration and pickling regeneration, etc.^51^. By controlling the SCR operating temperature, ABS deposition can be reduced, and the deposited ABS can be reduced by heating up. Zhang et al. indicated that online heating to control the temperature and strengthening soot blowing have a certain effect on the recovery of catalyst activity^52^.

The regeneration temperature of the catalyst is the key factor, the regeneration effect is very good at 350℃.The NH_3_-TPD on the sulfur poisoning catalyst found that the thermal regeneration of the catalyst can increase its adsorption of NH_3_. This change comes from the metal sulfate formed by the reaction of ABS with the catalyst. However, the metal sulfate consumes the active components of the catalyst, and the NH_3_ introduced during the thermal reduction regeneration reacts with the ABS decomposition products to generate ABS. The effect of continuing to cover the active sites is stronger than the ammonia adsorption sites increased by the metal sulfate. Therefore, the activity of the thermal regeneration and thermal reduction catalyst cannot be fully restored. The H_2_-TPR results also showed that the activity of thermal regeneration and thermal reduction regeneration W cannot be fully restored^4^.

Yang studied the formation and decomposition characteristics of ABS on the surface of the denitrification catalyst during the full load process. It is found that there are more deposits on the catalyst surface at 310℃, and the temperature can be increased to 330 ~ 350℃from the perspective of SCR operation to reduce the amount of deposits on the catalyst^18^. Since this research is one of the few full-load denitrification studies carried out at the power plant site, it is of great significance for the in-depth study of ABS under the background of full-load denitrification. It also means that the catalyst can be regenerated online by heating and other means.

Compared to offline or even online regeneration of catalysts, catalyst modification or the development of catalysts with ABS tolerance are more effective methods to deal with ABS problems.

### Air preheater

Initially, when ABS deposition occurred in the air preheater, it was found that steam soot blowing could not effectively solve the problem, and water washing was an effective way. However, too many washing times will not only produce a large amount of wastewater containing NH_3_, Fe, fly ash, etc., but also aggravate the corrosion of the heat exchange surface^34^. Therefore, in order to improve the cleaning efficiency, the online high-pressure water flushing of the air preheater as a technical measure to solve the blockage of the air preheater is gradually being accepted and adopted by power plants^53^.

Cai et al. put forward more practical suggestions for the ABS problem of air preheater: The flue gas bypass increases the flue gas temperature during low-load denitrification, adds a catalyst bed when the SCR catalyst is used for two years, and improves the arrangement of the heat transfer elements in the rotor. For example, the height of the cold section element is increased above the ABS deposition area (900 mm) and there is a low load and a margin of 50 ~ 100 mm in winter, and the cold section layer is easily dredged, the waveform is improved from the perspective of the heat transfer element to reduce the energy dissipation rate of the soot blowing and cleaning medium, or the enamel element is used to increase the smoothness. Replace the practice of blindly increasing soot blowing pressure and frequency with the reasonable use of dual medium soot blower and water flushing. The cold end heat transfer element is heightened so that the cold section includes the sulfuric acid corrosion zone and the ammonium bisulfate deposition zone, combining the intermediate layer and the cold section layer. The approach of the preheater supplier is to improve the waveform of the preheater, so that the energy dissipation of the soot blowing and cleaning medium in the heat transfer element is slowed, and the heat exchange element is selected according to the wet degree condation. Using an enamel surface to increase the surface finish and make it easy to remove the ash^54^. The above recommendations cover most specific ABS control methods.

Chen analyzed the influence of waveform and material on heat transfer performance, the influence of waveform on resistance, and the influence of air preheater temperature and resistance on air leakage rate. Discuss the influence of various influencing factors on the overall energy saving effect, and provide reference for solving the problems of air preheater blockage, corrosion and increased air leakage rate. And Chen reported that it can be washed with water at high exhaust temperature. The flushing water atomization evaporation equivalent to the steam flow reduces the risk of jamming and tripping caused by excessive water cooling and deformation of the air preheater^55^.

Lu et al. found that adding alkaline substances and increasing the porosity of fly ash can effectively reduce the deposition of ABS by studying the deposition of ABS adhering fly ash in the air preheater^39^.

### Precursors control

Zheng found through experiments that increasing the concentration of reactants can make the initial deposition smoke temperature of the air preheater ABS approach the average deposition smoke temperature. This showed that increasing the concentration of reactants can narrow the deposition temperature range of the air preheater, make the deposition more concentrated, and cause greater harm^20^. The industry has carried out extensive research on the control of the precursors of ABS formation and believed that the control of the precursors is an effective method to control ABS^56^.

Zhang summarized the methods of removing SO_3_ by alkali absorbent, WESP(Wet electrostatic precipitator), etc^27^. According to the position of the ABS control method, the methods of spraying alkali absorbent, blending low-sulfur coal, reducing excess air coefficient, optimizing ammonia injection grille and flue gas flow field are summarized. As far as ABS generation is concerned, it is mainly generated in SCR and air preheater. WESP arranged downstream of the air preheater can remove part of SO_3_ but cannot reduce the generation of ABS. WESP is not useless for ABS. Although it cannot reduce the generation of ABS in SCR and air preheater, it can reduce the emission of fine particles to a certain extent.

Hu proposed a method to optimize ammonia injection based on the uniformity of ammonia escape concentration field to reduce ammonia escape. The test results show that the optimization of ammonia injection can reduce NH_3_/NOx while ensuring the total denitrification efficiency through the adjustment of ammonia injection. Not only reduces ammonia consumption, but also reduces ammonia escape^57^. Yang analyzed the feasibility of full-load denitrification and found that ABS deposition on the catalyst surface is the result of the dual effects of temperature and concentration. It is pointed out that under the condition of ensuring a certain denitrification efficiency, reducing the amount of ammonia injection and ensuring a lower ammonia escape is an effective method to achieve full-load denitrification. At the same time, when the SCR inlet flue gas temperature is low (below 320℃), strengthening the purge of the catalyst area and the air preheater area can reduce the accumulation of ABS on the catalyst surface^18^.

Field operating experience showed that controlling ammonia escape could reduce ABS deposition. However, the ABS in the air pre-reactor may be generated by the reaction of NH_3_, SO_2_/SO_3_, and H_2_O in the air pre-reactor or migrated from the ABS in the SCR reactor. Therefore, by controlling the escape of ammonia to eliminate the problem of ABS deposition in the air preheater, it is also necessary to determine the proportion of ABS and SCR generated in the air preheater that migrate to the ABS of the air preheater.

Precursor control is from multiple angles such as ammonia injection concentration feedback and precise control. Optimize the flow field and temperature field to improve the efficiency of SCR denitrification reaction and reduce ammonia escape; Reduce the formation of SO_3_ from the perspective of combustion, coal quality, and catalyst composition; Removal of SO_3_ before ABS formation or deposition causes severe impact.

### Discusses

The impact of ABS on catalysts, SCR denitrification, air preheater, fly ash, particulate matter emissions, etc. can be divided into four processes including the formation, migration (volatility and decomposition), deposition and production of ABS. The regulation and control of ABS can achieve the best effect by take care of the above four processes. The formation of ABS can be reduced through precursors control such as alkali injection deamination, oxidative deamination between SCR and air preheater, desulfurization wastewater flue evaporation or low-temperature concentration coupled deamination, and appropriate SO_3_ removal methods. Promote migration or reduce deposition of ABS through temperature control. The generated ABS can only be passively adjusted to improve the tolerance of ABS's impact position. Such as SCR optimization of flue gas flow field and optimization of ammonia injection, development of catalysts with ABS tolerance, optimization of soot blowing methods in air preheaters, and use of enamel materials to reduce the impact of ABS viscosity, etc.

Research on the mechanism of the influence of catalysts on the decomposition behavior of ABS can lay a theoretical foundation or point out the direction for reducing ABS deposition and promoting ABS decomposition through catalyst modification. The research and development of wide-load denitrification catalyst with anti-ABS clogging ability has made certain achievements. The research in this direction is an important basis for realizing the transformation of denitrification flexibility. In addition, the existing researches mostly separates SCR and air preheater or studies the influence of ABS on SCR or the influence of ABS on air preheater. The precursors regulation and control ABS scheme could be clarified and optimized by taking SCR and air preheater as a whole to study the migration and deposition behavior of ABS. This is the research foundation of air preheater for ABS control, and it will also be the new research direction of ABS in air preheater.

In general, the research and development of SCR catalysts with wide temperature window and resistance to ABS poisoning, online activity recovery of catalysts or regeneration after poisoning, improvement of air preheater structure, optimization of soot blowing methods and precursors control are the fundamental measures to solve the ABS problem .

## Conclusions

1. The collection and detection of ABS-related substances is the prerequisite for studying the properties of ABS formation, deposition, decomposition, and migration. Selection of appropriate ABS collection and testing methods is a necessary condition to ensure the accuracy and correctness of the research. Increasing the condensation contact area can improve ABS collection effect.
2. ABS has a dual effect on denitrification and is related to temperature and ABS deposited amount. In general, the formation of ABS will reduce the denitrification efficiency when the temperature is low, and it will increase the denitrification efficiency when the temperature is high. The denitrification efficiency may be improved when ABS deposited amount is small, but when ABS deposited amount is large, the denitrification efficiency may be reduced. The reason for ABS influences on the denitrification efficiency of SCR is the specific surface area, pore volume, redox, acidity and other properties of the catalyst, and the NH_4_^+^ in ABS can react with NO to improve the denitrification efficiency.
3. ABS deposition causes an increase in the operating pressure difference of the air preheater and a decrease in heat transfer efficiency. The reaction of ABS with fly ash reduces the specific resistance of fly ash, which is beneficial to improve the efficiency of ESP(Electrostatic precipitator) dust removal, but the increased adhesion of fly ash makes it difficult to clean the electrode plate. The generation of ABS increases the concentration of fine particles at the SCR outlet, and some fine particles may penetrate the dust removal equipment and cause fine particles increase in the exhaust flue gas. ABS will also have a certain impact on the properties of fly ash.
4. The influence of ABS can be controlled from SCR operating conditions, catalyst modification, catalyst regeneration, modification and flushing optimization of air preheater, and precursors control. Precursors control, catalyst modification, or development of catalysts with low temperature denitrification activity and ABS tolerance are more fundamental methods for ABS regulation and control.
